# The impact of legislative regulation on animal epidemic prevention and control input: evidence from 13 main provinces of pig production in China

**DOI:** 10.3389/fvets.2025.1534046

**Published:** 2025-06-18

**Authors:** Wei Liu, Jianping Tao

**Affiliations:** ^1^College of Economics and Management, Huazhong Agricultural University, Wuhan, China; ^2^Hubei Rural Development Research Center, Huazhong Agricultural University, Wuhan, China

**Keywords:** legislative regulation, laws and regulations on pig epidemic prevention and control, animal epidemic prevention and control input, pig farmer, legal hierarchy, law enforcement practice

## Abstract

**Introduction:**

The issue of animal epidemic prevention and control has gained significant attention. Regulating and incentivizing farmers' animal epidemic prevention behaviors is vital for safeguarding national biosecurity. Previous studies have focused on the importance of animal disease prevention and control legislation but not examined the incentive of animal epidemic prevention behavior from the perspective of legislation. This study investigates the relationship between legislative regulation and farmers' animal epidemic prevention input, generating critical evidence for refining China's animal epidemic control framework and advancing the high-quality development of animal husbandry.

**Methods:**

Using balanced panel data from 13 main pig-breeding provinces in China from 2006 to 2022, this study employs the Feasible Generalized Least Squares (FGLS) method to: (1) evaluate the impact of legislative regulation on pig farmers' animal epidemic prevention and control input; (2) investigate the changes in epidemic prevention and control input of pig farmers of different scales and the differences in the effects of laws and regulations of different legal hierarchies, and (3) examine the impact of law enforcement practices on the effect of textual legislation.

**Results:**

Legislative regulation significantly increases animal epidemic prevention and control input, with the strongest effect on medium-scale farmers and no effect on large-scale farmers. The input-enhancing effect varies across laws and regulations of different legal hierarchies, with descending order: local administrative rules, central-level administrative regulations and divisional regulations, and local regulations. Heterogeneity analysis indicates that this input-enhancing effect of legislative regulation is only pronounced in regions with higher law enforcement on animal epidemic prevention and control.

**Discussion:**

This study can also provide important inspiration for other developing countries. Governments should intensify legal literacy initiatives, enhance farmers' regulatory awareness, implement regionally differentiated prevention measures, strengthen adaptive enforcement capacities, and ultimately realizing synergistic welfare gains across economic, biosecurity, and animal wellbeing domains.

## 1 Introduction

“One world, one health”. The realization of biosecurity is the common vision of all countries in the world. Outbreaks of animal diseases such as bird flu, blue ear disease, and African swine fever pose a serious threat to animal husbandry development, food security, and human health (1, 2), and the possibility of animal transmission has not been ruled out in the global COVID-19 epidemic (3). In 2020, Xi Jinping, general secretary of the Communist Party of China Central Committee, stressed that “we should strengthen the protection of rule of law in public health and comprehensively intensify and improve the construction of relevant laws and regulations in the field of public health” at the 12th meeting of the Communist Party of China Central Committee for Comprehensively Deepening Reform. The construction of laws and regulations is an important aspect of the legalization of the prevention and control of animal epidemics (4), which institutionalizes the prevention and control policy of animal epidemics through legalization, making it more stable, continuous, and authoritative. Since the promulgation of the Animal Epidemic Prevention Law in 1997, hundreds of laws and regulations on animal disease prevention have been passed by the Chinese and provincial legislatures, and the Agricultural Law (amended in 2012), the Biosecurity Law (enforced in 2021), and the Animal Epidemic Prevention Law (revised in 2021) have come into force. These laws and regulations cover implementation measures, technical specifications, disease classification, legal liability, and other aspects and stipulate the responsibility of livestock producers for animal epidemic prevention. Farmers play a crucial role in biosecurity as they are the first to notice changes in the health or productivity of their livestock and are on the front lines of animal epidemic prevention (5), determining the effectiveness of the government's animal disease control system. How to effectively motivate farmers to prevent and control animal epidemics? Particularly, can legislative regulation increase farmers' animal epidemic prevention and control input (referred to as “animal epidemic prevention input”)? The scientific answers to the above questions have important theoretical value and practical reference significance for improving China's animal epidemic prevention and control policies and promoting the high-quality development of animal husbandry.

The academic literature on the factors affecting the prevention and control of animal epidemics in farmers is abundant. There have been studies on various aspects of animal epidemic prevention and control, such as vaccine injection (6), decision-making on the resource treatment of sick and dead pigs (7), and the adoption of biological isolation measures such as bird control, rat control, vehicle disinfection, and personnel disinfection (8, 9). In addition, some scholars have measured farmers' animal epidemic prevention behavior by the number of epidemic prevention measures (10) and medical epidemic prevention expenditure (11). The influence of farmers' social and demographic characteristics such as gender, age, and education level and breeding characteristics such as income structure, breeding scale and breeding years, disease risk cognition, knowledge of epidemic prevention measures, and policy cognition on animal disease prevention and control behavior has been widely recognized (12, 13). Due to the large externality of animal disease prevention and control, government intervention is of great significance for animal disease prevention and control (14). Using mathematical modeling, Tian et al. (15) found that increasing punishment could significantly increase the risk faced by farmers in concealing the epidemic and thus drive them to report the epidemic. Si et al. (16) believed that the withdrawal period supervision mainly forced farmers to regulate veterinary drug use by improving their perceived level of loss risk. Some scholars believe that regulatory policies and subsidy policies work in a similar way where they both improve production behaviors by affecting expected revenue and expected cost (17). The government can also reduce the time and labor costs required for farmers to verify disease conditions and implement animal epidemic prevention measures by publicly providing animal disease information and epidemic prevention technical assistance (18, 70).

It is worth pointing out that laws and regulations not only represent command-and-control government regulation but also provide a legal basis and guarantee for administrative penalties and rights compensation, which is the key to promoting the normalization of animal epidemic prevention and control. Qin (19) discussed the compatibility of the newly revised Biosecurity Law with existing legislation on animal disease prevention and control. Yu et al. (20) took the livestock forbidden area policy in the Regulation on the Prevention and Control of Pollution from Large-scale Breeding of Livestock and Poultry implemented by China in 2014 as a natural experiment and found that the policy improved environmental standards of the livestock industry and forced farmers to make resource utilization of livestock and poultry manure. However, there are still some scholars who doubt the importance of textual legislation in China (21) and believe that China's textual legislation generally has the problem of “incomplete implementation” (22).

The existing research has important theoretical value and practical significance for improving the animal epidemic prevention behavior of farmers, but there is still some room for expansion. First, animal epidemic prevention behavior includes all aspects of preventing pathogens from entering and spreading among animals (23). However, existing literature pays more attention to one link of animal epidemic prevention or one specific epidemic prevention technology and lacks analysis of the comprehensive performance of various epidemic prevention behaviors of farmers under epidemic risk^1^. Second, the existing literature has focused on the importance of animal disease prevention and control legislation but has not examined the incentive of animal epidemic prevention behavior from the perspective of legislation. In addition, domestic and foreign scholars mainly regard legislative regulation as command-and-control regulation. However, any law or regulation not merely contains a kind of policy tool but often comes with financial support and regulatory measures. Therefore, it is imperative to study the entire animal disease prevention and control policy as a system. Third, current studies related to animal epidemic prevention and control policies mainly focus on policy optimization, or the inclusion of this policy as a control variable. The heterogeneity of animal epidemic prevention and control policy on animal epidemic prevention and control behavior remains to be further explored.

Introducing the textual legislative regulation of pig epidemic prevention and control laws and regulations into the research of incentives for farmers' animal epidemic prevention and control behavior, this study employs the balanced panel data of 13 provinces with advantageous pig breeding in China from 2006 to 2022 to analyze the epidemic prevention promotion effect of legislative regulation, to investigate the changes in epidemic prevention and control input of pig farmers of different scales and the differences in the effects of laws and regulations of different legal hierarchies, and to examine the impact of law enforcement practice on the effect of textual legislation. This article aims to provide a reference for incentivizing farmers' epidemic prevention and control decision-making, improving the animal epidemic prevention and control policy, and boosting the high-quality development of animal husbandry. The 13 provinces with advantageous pig breeding are selected on the following basis. The 10 provinces (autonomous regions), namely, Sichuan, Hunan, Henan, Shandong, Hebei, Guangdong, Guangxi, Hubei, Jiangsu, and Anhui, accounted for approximately 65% of the total amount of pigs slaughtered^2^ and are the major pig producing regions. Taking into account the spatial transfer trend of “southern pigs moving northward”, the three provinces of Heilongjiang, Jilin, and Liaoning are included in the analysis.

## 2 Theoretical framework

In this study, legislative regulation refers to textual legislation regulation on pig epidemic prevention and control, which is measured by the number of laws and regulations on pig epidemic prevention and control. The Constitution of the People's Republic of China declares that the legislative body includes central and local levels. So, the laws and regulations on pig epidemic prevention and control refer to the laws and regulations promulgated by the central and local governments with the aim of promoting the development of the pig industry, ensuring public health safety and human health, and acting on the animal epidemic prevention and control behavior of pig breeding individuals or organizations.

The theoretical basis that the laws and regulations on pig epidemic prevention and control affect animal epidemic prevention input is mainly the externality theory. The externalities of pig epidemic prevention and control consist of two aspects: negative externalities of not implementing epidemic prevention and control measures and positive externalities of implementing epidemic prevention and control measures. In terms of negative externalities, if farmers do not take measures to prevent and control epidemics in pig breeding, it will accelerate the spread of the epidemic and cause public health and food health problems. All nearby residents, including other pig farmers who actively prevent epidemics, will be affected by it. Thus, the epidemic prevention cost that should be borne by individual pig farmers is shared by all, and the marginal private cost is less than the marginal social cost. Due to economic factors, insufficient epidemic prevention often occurs. In terms of positive externalities, when farmers take animal epidemic prevention and control measures and get epidemic prevention benefits, surrounding farmers and even the whole society can enjoy the benefits of reducing animal epidemic risk for free, resulting in the phenomenon of “free riding”. The marginal private benefit is lower than the marginal social benefit, which makes them less active in adopting epidemic prevention and control measures. Government intervention is an important way to solve the externality problem. Appropriate government policies cause the marginal private benefit and marginal social benefit to gradually converge to the equilibrium point, thus internalizing the externality (24).

There is no doubt that the law is irresistible and mandatory. According to this feature of law, many researchers have used the number of decrees issued by the government to measure compulsory government regulation (25, 26). Meanwhile, the law also plays a role in information dissemination and guidance (27). Legislative regulation can affect animal epidemic prevention input in two aspects: information dissemination and behavior deterrence. From the perspective of information dissemination, the central and local governments are the main information dissemination sources of laws and regulations on pig epidemic prevention and control. These laws and regulations are seen as important biosecurity information carriers (28), but their content is often obscure as formal institutional texts. According to compensatory control theory, when a law or a regulation on pig epidemic prevention and control is promulgated, farmers lack understanding and awareness of it, and their sense of control will decrease (29). Motivated to compensate for their sense of control, farmers will increase attention to information related to pig epidemic prevention and control laws and regulations (30). Information is the basis of risk judgment and individual decisions on preventive behavior (31). Information attention is an important component of farmers' information awareness, which emphasizes the subjective initiative of the information subject and directly affects farmers' decision-making (32). Therefore, the more attention farmers pay to epidemic prevention information, the more information they receive and search, the stronger their awareness of epidemic prevention, and the more consciously they will carry out animal epidemic prevention measures.

From the perspective of behavior deterrence, laws and regulations grant administrative punishment rights to the administrative department of the Ministry of Agricultural and Rural Affairs. They can impose administrative punishment such as warning, criticism, fines, revocation of licenses, and suspension of production and business on subjects who act inappropriately in pig epidemic prevention and control practices pursuant to the law, and they can even refer cases and personnel involved in disciplinary offenses to the judicial authorities to pursue legal liability,^3^ which not only punishes violators but also has a deterrent effect on other farming subjects. Becker (33) believed that the certainty and the severity of punishment deter crime. The intensive promulgation of laws and regulations on pig epidemic prevention and control will not only convey to farmers that regulation on pig epidemic prevention and control is becoming more frequent but will also raise farmers' attention to the administrative penalties for epidemic prevention violations and make them perceive higher violation costs. These expected ex-post costs will act as ex-ante incentives (34), driving farmers to adjust their epidemic prevention decisions. As a result, the deterrent effect of pig epidemic prevention and control laws and regulations will be significantly enhanced, and pig farmers will be more likely to actively prevent the epidemic and increase epidemic prevention input. Based on this, the following hypothesis is proposed.

H: Legislative regulation on pig epidemic prevention and control can increase farmers' animal epidemic prevention input. Legislative regulation on pig epidemic prevention and control gradually balances the marginal private benefit and marginal social benefit of farmers, thus improving the enthusiasm for epidemic prevention and control and affecting their animal epidemic prevention and control behavior.

## 3 Sample and empirical strategy

### 3.1 Data sources

The data used in the study are a balanced panel data of 13 provinces with advantageous pig breeding in China from 2006 to 2022 (*N* = 221). We obtain data on animal epidemic prevention and control input of pig breeding from the National Compilation of Information on Cost and Benefit of Agricultural Products,^4^ with some missing data determined by interpolation. The laws and regulations on pig epidemic prevention and control data are derived from the PKULAW Database,^5^ Data on the education of rural households are from the China Population and Employment Statistical Yearbook.^6^ Data on the proportion of wages in the disposable income of rural households are from the China Yearbook of Rural Household Survey,^7^ the China Yearbook of Household Survey,^8^ and provincial statistical yearbooks. Data on pig market price, the number of employees in township animal husbandry and veterinary stations, and the number of pig breeding households are obtained from the China Animal Husbandry and Veterinary Yearbook^9^ and China Animal Husbandry Information Network.^10^ Data on slaughtered pigs, gross domestic product (GDP), road, railway, inland waterway mileage, and per capita disposable income of rural households are from the China Statistical Yearbook.^11^ We also make use of the Official Veterinary Bulletin^12^ published by the Ministry of Agriculture and Rural Affairs of the People's Republic of China to find pig death and cull data. Sample data processing and regression analysis are performed using STATA 16.0.

Pig farmers, as an important subject in the development of the pig industry, are the primary object of scholars' research on the pig industry. The use of macro-level farm household data to measure the behavior and endowment characteristics of micro-farmers is well documented. Based on the number of farms of different scales in 30 provinces, the transition probability of pig breeding scale structure is measured (25). Rural residents' education level is usually used to characterize the education level of pig farmers when studying the determinants of pig industry development (35, 36). The number of research and development personnel at the provincial level can be an indicator of the scientific and technological input in the hog industry (37).

Compared with previous studies, the data used in the study are from open statistical data published by China's National Bureau of Statistics (CNBS). It reduces systematic errors and keeps the core indicators of the data unchanged and comparable (38). At the same time, the data cover 13 provinces with advantageous pig breeding in China, which can represent the overall level of legislative regulation on pig epidemic prevention and control.

### 3.2 Data and sample description

#### 3.2.1 Dependent variable-animal epidemic prevention and control input (Input)

The expenditure on animal medical and epidemic prevention is part of the production cost. Although it increases the total cost, it can reduce the risk of epidemic in pigs and guarantee stable long-term returns for farmers (39, 40). Animal epidemic prevention and control behavior is a collection of a series of epidemic prevention measures. Therefore, when exploring the promotion effect of legislative regulation on animal epidemic prevention input, one should not only focus on farmers' input in a specific epidemic prevention measure but also examine the overall epidemic prevention input as a priority. In addition, protecting susceptible animals and treating sick animals are the most common biosecurity measures applied by farmers (23, 41), and the elements involved include vaccines, veterinary drugs, antibiotics, disinfection drugs, and other epidemic prevention and treatment substances, with inconsistent measurement units and product types. Using expenditure on medical and epidemic prevention as a measure of animal epidemic prevention and control input can avoid the aforementioned issue. The natural logarithm of the average expenditure on medical and epidemic prevention of pig farmers of different scales (scattered farmers and small-, medium-, and large-scale farmers) is chosen as a proxy variable for animal epidemic prevention and control input.

#### 3.2.2 Core independent variable-legislative regulation (LR)

Drawing on Mo et al. (42), the number of laws and regulations on pig epidemic prevention and control at the central level and the local level from 2006 to 2022 is obtained from the PKULAW Database, a professional authoritative database of policies and regulations in China. The laws and regulations are searched by “animal epidemic + pig” and counted yearly.^13^ On this basis, the number of laws and regulations at local and central levels retrieved in the corresponding year is summed up to obtain the number (flow) of new laws and regulations on pig epidemic prevention and control in province i in year t. Since a piece of legislation is valid for more than 1 year, to examine the level of legislative regulation on pig epidemic prevention and control in province i in year t, it is necessary to obtain the sum (stock) of all laws and regulations in force in province i in year t. Therefore, the natural logarithm of the sum of laws and regulations on pig disease prevention and control is taken as a proxy variable for legislative regulation.^14^

#### 3.2.3 Control variables

Drawing on the existing literature on the influence of farmers' animal epidemic prevention behavior, the following control variables are introduced.

##### 3.2.3.1 Education (Edu)

More educated farmers tend to adopt production techniques and management practices that meet biosecurity and institutional needs (23). As there is a lag in the effect of education on farmers' behavior (43, 44), a lagging education is used for the regression^15^. Education is calculated by Equation 1.


(1) $$Edu={\left(\frac{PP\times 6+JP\times 9+SP\times 12+TP\times 16}{TTP}\right)}_{t-1}$$

where PP is the rural population with primary education. JP is the rural population with junior secondary education. SP is the rural population with senior secondary education. TP is the rural population with tertiary and above education. TTP is the total rural population aged 6 years and above.

##### 3.2.3.2 Income structure (IS)

Farmers' income structure can reflect the degree of farmers' dependence on pig farming, which is an important factor influencing farmers' biosecurity behavior (18). Instead, Zhang and Zhang (45) argued that the higher the proportion of farm income, the more likely farmers are to increase epidemic risk exposure and adopt short-sighted behavior in management practices in pursuit of low-cost and high returns. The proportion of wages in the disposable income of rural households is chosen as a measure of income structure.

##### 3.2.3.3 Pig market price (Price)

The market price is the wind vane of the development of the pig industry. In regions with higher pig prices, the economic development is relatively better, the comprehensive quality of farmers is higher, and they are more willing to comply with the requirements of animal disease prevention laws and regulations on epidemic prevention. In addition, given the positive correlation between pig prices and expected return, the higher the pig price, the higher the farmers' expected return, and they will increase biosecurity precautions to prevent pigs from being infected with the virus (46). As short- and medium-term market prices are more exogenous than long-term market prices and may influence farmers' behavior (25), the annual average pig price, derived from the monthly pig prices, is used as a proxy variable for the pig market price.

##### 3.2.3.4 Economic development (Dgdp)

A sound financial guarantee mechanism is an important prerequisite for animal disease prevention and control work (47). The higher the level of regional economic development, the better the financial guarantee mechanism for animal disease prevention and control, and the more the government invests in the construction of animal disease prevention and control infrastructure, which is more likely to improve farmers' enthusiasm for disease prevention and control. This variable is characterized by the natural logarithm of the deflated GDP.

##### 3.2.3.5 Scale breeding (Scale)

Scale breeding is the developing direction of the modern pig industry, which to a certain extent reflects the continuous improvement of the technical level (48). The higher the level of scale breeding, the higher the level of farming technology, and the greater the likelihood that farmers increase their efforts in epidemic prevention and control. From another perspective, the degree of pig scale breeding depends on the number of scale farms (households). The scale farms (households) have rich knowledge reserves of animal epidemic prevention and control, strong production capital, and high awareness of biosecurity, so they can establish a complete biosecurity system by introducing advanced epidemic prevention and control technology and equipment (49, 50). Following the practice of Yang and Wang (49), we use the percentage of scale farms (households) of 500 or more pigs slaughtered annually in the total number of farms (households) to measure it.

##### 3.2.3.6 Convenience of technical service (Conv)

The impact of technical service support on the adoption of biosecurity behavior has also attracted scholars' attention (45). Improved transport conditions facilitate farmers to seek technical guidance from service organizations such as professional veterinarians, animal hospitals, or universities. Referring to Huang et al. (25), the natural logarithm of the sum of road, railway, and inland waterway mileage in each province is used to measure the variable.

##### 3.2.3.7 Pig epidemic shock (Epi)

The more severe the epidemic shock, the more farmers invest in epidemic prevention (51). Empirical studies (49, 50) demonstrate that farmers' animal epidemic prevention and control behaviors exhibit time-dependent adjustments, primarily shaped by their retrospective evaluation of prior outbreak severity. The historical epidemic informs future biosecurity decisions. Pig epidemic shock is calculated by Equation 2.


(2) $$Epi={\left(\ln(\frac{Death+Culling}{Slaughter}+1)\right)}_{t-1}$$

where Death and Culling are the number of pig deaths and forced culls caused by nine pig epidemics, respectively. Slaughter is the number of pigs slaughtered. To reduce the impact of outliers and heteroscedasticity, we take the natural logarithm of this variable. The inclusion of the “+1” inside the logarithm in Equation 2 is a mathematical necessity to handle non-negative variables that can be zero, ensuring that the argument of the logarithm is always positive, thus avoiding the undefined value problem. By adding 1, we are effectively shifting the distribution of the variable slightly to the right, which does not fundamentally alter the relationship between the variables but allows us to use the logarithmic transformation for our analysis.^16^

The descriptive statistics of the variables are presented in Table 1.

**Table 1:** Descriptive statistics.

**Variables**		**(1)**	**(2)**	**(3)**	**(4)**	**(5)**
		**N**	**Mean**	**SD**	**Min**	**Max**
Dependent variable	Input	221	2.9571	0.3810	1.8798	3.7906
Independent variable	LR_stock	221	5.8553	0.8693	3.3322	7.2086
	LR_flow	221	3.9395	0.3139	3.0910	5.2523
Control variables	Edu	221	7.6682	0.4869	4.0853	8.6538
	IS	221	0.3358	0.1107	0.0960	0.5750
	Price	221	15.9675	5.7679	6.5008	37.2033
	Dgdp	221	9.3287	0.5904	8.0792	10.5182
	Scale	221	1.3900	1.5468	0.0220	10.2450
	Conv	221	12.1584	0.3488	11.4015	12.9531
	Epi	221	0.3755	0.7569	0.0000	4.7980

To ensure data comparability, all variables denominated in currencies in the table are measured in 2006 constant prices.

### 3.3 Empirical strategy

To test the underlying relationship between legislative regulation and animal epidemic prevention and control input, the model is as defined in Equation 3.


(3) $$\begin{array}{l} Input_{it}=b_{0}+b_{1}LR_{it}+\overset{J}{\underset{i=1}{\sum }}w_{j}Control_{it}+\alpha_{i}+\gamma_{t}+\varepsilon_{it} \end{array}$$

where i denotes the region, and t represents the year. Input denotes animal epidemic prevention and control input. LR denotes the level of legislative regulation (flow/stock) on pig epidemic prevention and control. Suppose the coefficient b_1_ of the independent variable LR is significant and positive. In this case, it indicates that the promotion effect of legislative regulation on animal epidemic prevention and control input does exist. Control is a series of control variables affecting animal epidemic prevention and control input. α and γ are vectors of the province and year dummy variables that account for province and year fixed effects, and ε is the error term.

## 4 Empirical results and discussion

### 4.1 Effect of legislative regulation on animal epidemic prevention and control input

The results of the variance inflation factor (VIF) test for independent variables are shown in Table 2. It shows that the largest VIF value is 4.5400, much < 10. Therefore, multicollinearity is proved to be weak. This has reached a basis for the next regression analysis.

**Table 2:** Variance inflation factor (VIF).

**Variable**	**Dgdp**	**IS**	**LR_stock**	**Scale**	**Price**	**Conv**	**Edu**	**Epi**	**Mean**
VIF	4.5400	3.4000	2.7600	2.1600	2.1100	1.8900	1.4700	1.2100	
1/VIF	0.2200	0.2940	0.3630	0.4620	0.4740	0.5300	0.6810	0.8270	2.4400

There may be interactions between contemporaneous economic activity across regions, so we first perform a modified Wald test for between-group heteroscedasticity, a Woodridge test for within-group autocorrelation, and a Pesaran test for between-group contemporaneous correlation on the panel data. All three tests strongly reject the original hypothesis,^17^ indicating that the model developed has between-group heteroscedasticity, within-group autocorrelation, and between-group contemporaneous correlation. The feasible generalized least squares method (FGLS) is known to be more efficient than OLS in the presence of heteroscedasticity, and serial and/or cross-sectional correlation (52, 53). Therefore, we apply FGLS that allows different individual disturbance terms to be contemporaneously correlated and have different variances, while controlling for individual factors that do not vary over time and the effect of time trends.

Table 3 presents the results of the model. Whether LR is measured by the stock or the flow of the number of laws and regulations on pig epidemic prevention and control, its coefficients are all positive and statistically at a 1% confidence level. It indicates that legislative regulation can indeed increase animal epidemic prevention and control input, and the hypothesis is confirmed. This is consistent with previous studies. Both qualitative (39) and empirical analyses (51) show that government regulation has a positive impact on farmers' animal epidemic prevention and control behavior. First, the laws and regulations on pig epidemic prevention and control convey information on pig epidemic hazards, probability of occurrence, and epidemiological status to farmers, which will improve their perception of disease hazards. Second, the issue of pig epidemic prevention and control laws and regulations has aroused their information demand on epidemic prevention and control. Farmers take the initiative to acquire knowledge related to disease prevention and control technology, heighten awareness of disease prevention and control and production efficiency, and reduce uncertainty in behavioral decisions. In addition, the intensive introduction of laws and regulations on pig epidemic prevention and control has enhanced the deterrent effect on farmers' opportunism. It promotes the probability and penalty cost of farmers' violation of epidemic prevention and strengthens the punishment perception for epidemic prevention violations, which in turn motivates them to increase their epidemic prevention and control input.

**Table 3:** Baseline regression.

	**(1)**	**(2)**	**(3)**	**(4)**
**Variables**	**Input**	**Input**	**Input**	**Input**
LR_stock	0.2159^***^	0.1431^***^		
	(0.0283)	(0.0263)		
LR_flow			0.1462^***^	0.0970^***^
			(0.0201)	(0.0099)
Edu		−0.0010		0.0116^*^
		(0.0090)		(0.0068)
IS		−0.4001^***^		−0.1976^*^
		(0.1328)		(0.1181)
Price		0.0058^***^		0.0072^***^
		(0.0017)		(0.0012)
Dgdp		0.4909^***^		0.9255^***^
		(0.1006)		(0.0785)
Scale		0.0305^***^		0.0264^***^
		(0.0059)		(0.0051)
Conv		−0.1198		−0.1033
		(0.0792)		(0.0631)
Epi		0.0116^**^		0.0122^***^
		(0.0049)		(0.0040)
Constant	1.3853^***^	−0.9558	1.7495^***^	−4.5955^***^
	(0.1349)	(1.3305)	(0.1005)	(0.9625)
Province FE	Yes	Yes	Yes	Yes
Year FE	Yes	Yes	Yes	Yes
*N*	221	221	221	221

The standard errors are reported in the brackets. ^***^, ^**^, and ^*^ represent significance at the 1%, 5%, and 10% levels.

As shown in column (2) of Table 3, income structure has a significant negative effect on animal epidemic prevention and control input. It suggests that the higher the proportion of wages, the less farmers rely on pig breeding and are prone to neglect pig epidemic prevention and control. Pig market price exhibits a positive effect on animal epidemic prevention and control input. Generally speaking, the higher the pig price, the stronger the motive of farmers to hide pig disease or secretly sell sick and dead pigs for higher pay. Such behavior may result in farmers facing huge fines or even criminal penalties.^18^ So, even with rising pig prices, farmers will not risk legal limbo. Areas with higher prices are relatively more economically developed, and farmers in those areas are better qualified and more willing to comply with the requirements of animal disease prevention laws and regulations on epidemic prevention. Economic development promotes farmers' input in animal epidemic prevention and control. The higher the level of economic development, the more funds for animal epidemic prevention and control, which can create favorable conditions for farmers to prevent and control animal epidemics. In areas with a high level of scale breeding, farmers are more aware of biosafety and will be more cautious in increasing epidemic prevention input to avoid diseases in their pigs. Farmers in areas with severe pig epidemic shock have strong epidemic risk perceptions and are more active in animal epidemic prevention and control.

### 4.2 Addressing endogeneity and robustness check

#### 4.2.1 Addressing endogeneity

The possible existence of a “two-way causality problem” between legislative regulation and animal epidemic prevention and control input raises endogeneity concerns. Specifically, the intensity of legislative regulation on pig epidemic prevention and control may also be influenced by farmers' animal epidemic prevention and control behavior. The instrumental variable method (IV) is employed to address this issue (Table 4). We follow Zhang et al. (54) and use LR_iv as an instrumental variable for LR. LR_iv is measured by the mean value of legislative regulation intensity in neighboring provinces.^19^ As can be seen from columns (1) and (2), the F-value of the first stage is >16.38, and the *p*-value is 0.0002. It proves that this instrumental variable is valid and rejects the original hypothesis that there is no endogeneity problem. Column (2) demonstrates that the coefficient of LR_iv is still significant and positive, supporting that legislative regulation helps to enhance animal epidemic prevention and control input.

**Table 4:** Endogeneity and robustness check.

	**(1)**	**(2)**	**(3)**	**(4)**	**(5)**
	**First**	**Second**	**Replacing Y**	**Replacing X**	**Lagged X**
**Variables**	**LR_stock**	**Input**	**Input_p**	**Input**	**Input**
LR_iv	0.2157^***^				
	(0.0436)				
LR_stock		0.3423^***^	0.0157^*^		
		(0.0997)	(0.0090)		
LR_c				0.1429^***^	
				(0.0183)	
LR_stock_1					0.0271^*^
					(0.0164)
Constant			−0.4710^***^	0.1429^***^	−6.4388^***^
			(0.1013)	(0.0183)	(1.2802)
CV	Yes	Yes	Yes	Yes	Yes
Province FE	Yes	Yes	Yes	Yes	Yes
Year FE	Yes	Yes	Yes	Yes	Yes
*N*	221	221	221	221	208
R-squared	0.749				
F-value in the first stage	24.48				
*p*-value	0.0000				
Kleibergen-Paap rk LM statistic		13.665			
*p*-value		0.0002			
Kleibergen-Paap Wald rk F statistic		24.479			

Columns (1) and (2) are the results of IV. The standard errors are reported in the brackets. ^***^, ^**^, and ^*^ represent significance at the 1%, 5%, and 10% levels. CV represents the control variables. Yes represents all the control variables are added to the model. The regression coefficients presented are significant at the 1% or 10% level, while the regression coefficients not presented are significant at either the 1%, 5%, or 10% level.

#### 4.2.2 Robustness check

Robustness tests are conducted in the following aspects, and the specific results are shown in Table 4.

##### 4.2.2.1. Replacing the dependent variable

The percentage of expenditure on medical and epidemic prevention in per capita disposable income of rural households (Input_p) is selected to replace the dependent variable.

##### 4.2.2.2 Replacing the core independent variable

The natural logarithm of the number of legal entries on animal epidemic prevention and control (LR_c) from the China Legal Knowledge Database (CLKD)^20^ is used as a proxy for legislative regulation.

##### 4.2.2.3 The one-period lagged core independent variable

Given the lag of legislative regulation on pig epidemic prevention and control^21^, we take a lagged period for the core independent variable (LR_stock_1) in regression.

All the regression findings, which correspond to columns (3)–(5) in Table 4, are consistent with the claim that the strengthening of legislative regulation has significantly increased animal epidemic prevention and control input.

### 4.3 Differential performance of farmers of different farm scales

Studies conducted in the UK (55) and Indonesia (56) found that broiler production systems regulated under the same law differed in biosecurity performance, which attributed to differences in farm characteristics. So, how would the animal epidemic prevention and control behavior of pig farmers of different farm scales in China differ under the legislative regulation? Therefore, this study examines the changes in epidemic prevention and control input of large-scale farmers (annual slaughter of 10,000 head and above), medium-scale farmers (annual slaughter of 3,000–9,999 head), small-scale farmers (annual slaughter of 500–2,999 head), and free-range farmers (annual slaughter of 499 and below), separately.

Table 5 shows that the strengthening of legislative regulation has a significant contribution to the epidemic prevention and control input of medium-scale farmers, small-scale farmers, and free-range farmers, and the incentive effect decreases sequentially. However, legislative regulation has no significant impact on large-scale farmers. Medium-scale farmers may expand to large-scale farmers to implement the scale operation, and their requirement for animal epidemic prevention and control will be more stringent. Small-scale farmers are a high-risk sector (57, 58), they have less access to information and knowledge on biosecurity practices (56), and their biosecurity awareness and epidemic prevention capacity are yet to be improved. Motivated by animal epidemic prevention laws and regulations, they expand the scale of epidemic prevention input, which also fits the view that the smaller the farm scale, the more sensitive the farmers are to policy (25). Under continuous strengthening of legislative regulation on animal epidemic prevention and control, the stable policy expectation of free-range farmers with less fixed investment and free access to the market has evolved into stable benefit expectations, which can encourage their animal epidemic prevention and control input. Large-scale farmers usually adopt standardized management modes, with normative biosecurity management and high levels of epidemic prevention and control, and thus, they are not greatly affected by animal epidemic prevention laws and regulations. However, the possibility of a higher probability of epidemic transmission in scale farms due to animals being housed nearby should not be ignored (59).

**Table 5:** Differential performance of farmers of different farm scales.

	**(1)**	**(2)**	**(3)**	**(4)**
**Variables**	**Large-scale**	**Medium-scale**	**Small-scale**	**Free-range**
LR_stock	−0.0211	0.2315^***^	0.1932^***^	0.1381^***^
	(0.0277)	(0.0124)	(0.0227)	(0.0229)
Constant	−4.7344^***^	−4.8202^***^	−0.5003	2.6063^*^
	(1.3770)	(1.0165)	(1.4300)	(1.3395)
CV	Yes	Yes	Yes	Yes
Province FE	Yes	Yes	Yes	Yes
Year FE	Yes	Yes	Yes	Yes
*N*	221	221	221	221

The standard errors are reported in the brackets. ^***^, ^**^, and ^*^ represent significance at the 1%, 5%, and 10% levels. CV represents the control variables. Yes represents all the control variables are added to the model. The regression coefficients presented are significant at the 1% or 10% level, while the regression coefficients not presented are significant at either the 1%, 5%, or 10% level.

### 4.4 Effect of different legal hierarchy

The animal epidemic prevention legislation contains law, administrative regulation, divisional regulation, local regulation, local government rule, local normative document, and local working document. The association between legislative regulation and animal epidemic prevention and control input is expected to vary with the legal hierarchy. Laws and regulations of different legal hierarchies issued by different subjects have differences in liability, supervision, and applicability. Initiatives that draw on locally situated practices and knowledge of disease are more likely to have an impact on biosecurity (60). Insight into the association between laws and regulations of different legal hierarchies and animal epidemic prevention and control input is, therefore, useful.

Table 6 gives the results of the effect of central (central-level administrative regulations and divisional regulations), local regulations, and local administrative rules,^22^ respectively. Positive effects of these laws and regulations on animal epidemic prevention and control input are observed, with descending order: local administrative rules, central, and local regulations.

**Table 6:** Effect of different legal hierarchy.

	**(1)**	**(2)**	**(3)**
	**Central**	**Local regulation**	**Local administrative rule**
**Variables**	**Input**	**Input**	**Input**
LR_stock	0.1062^***^	0.0036	0.1720^***^
	(0.0271)	(0.0118)	(0.0206)
Constant	−1.6370	−3.9401^***^	−1.3278
	(1.3702)	(1.2040)	(1.1459)
Province FE	Yes	Yes	Yes
Year FE	Yes	Yes	Yes
CV	Yes	Yes	Yes
*N*	221	221	221

The LR_stock of columns (1)–(3) is central-level administrative regulations and divisional regulations, local regulations, and local administrative rules, respectively. The standard errors are reported in the brackets. ^***^, ^**^, and ^*^ represent significance at the 1%, 5%, and 10% levels. CV represents the control variables. Yes represents all the control variables are added to the model. The regression coefficients presented are significant at the 1% level, while the regression coefficients not presented are significant at either the 1%, 5%, or 10% level.

Although the legal hierarchy of local administrative rules is lower than that of local regulations, the epidemic prevention promotion effect of administrative rules is better than that of local regulations. Local administrative rules on animal epidemic prevention are normative documents promulgated by local governments according to the actual situation and needs (61, 62). They mainly reflect the local government's interests and preferences, and they may be implemented far more efficiently than local regulations in practice. It can be concluded that the local administrative rules on animal epidemic prevention and control have substantial incentive and constraint effects on farmers' pig disease prevention and control behaviors and improve their epidemic prevention and control input. Central-level administrative regulations and divisional regulations are the programmatic documents for local government and departmental administration at all levels (63), and they play an important guiding role in the management of local animal disease prevention and control. In the Internet era, one of the criteria for farmers to quickly select information is whether it is published by official media. When laws and regulations at the central level are introduced, heavyweight central and local official media will report and publicly interpret them several times. More importantly, Chinese citizens give priority to policy signals from the central government compared to those from local governments (69).

### 4.5 Heterogeneity analysis on animal epidemic prevention law enforcement practices

Textual legislation and law enforcement practice are important guarantees for advancing law-based governance. In a situation where textual legislation in China is generally “not fully enforced” in practice (22), the incentive effect of legislative regulation on farmers depends not only on the promulgation of textual legislation but also on the intensity of actual law enforcement. Insight into the differences in the effect of legislative regulation on animal epidemic prevention and control input in areas with different intensities of law enforcement is, therefore, necessary.

According to the Administrative Measures for Rural Animal Husbandry and Veterinary Stations, the primary duties of township animal husbandry and veterinary stations include propagating and implementing guidelines, policies, laws, and regulations for the development of animal husbandry, and supervising animal epidemic prevention of units and individuals that engage in raising or marketing of animals, or production or marketing of animal products. The role of grassroots animal husbandry and veterinary stations in the construction of the “bottom of the net” is crucial in opening up the “last mile” of epidemic prevention.

In consideration of the data availability, the ratio of the number of employees in township animal husbandry and veterinary stations to the number of pig farming households was chosen to measure the intensity of law enforcement. Using the median of law enforcement intensity as the dividing criterion, we divided the sample into two subsamples: areas with greater than the median law enforcement (high law enforcement) and areas with less than the median law enforcement (low law enforcement). The statistical result shows that the average number of township animal husbandry and veterinary staff per 1,000 households in regions with high law enforcement is 11, which is higher than the sample with low law enforcement (3 persons per 1,000 households). Larger regions often possess more complex livestock supply chains, potentially diluting regulatory oversight through fragmented implementation. In the prevention and control of animal epidemic in China, resource-thinning risk in large-scale regions, where fixed enforcement resources are spread thinly across an extensive population, potentially undermines regulatory efficacy.

The impact of law enforcement on textual legislation is displayed in Table 7. In regions with high law enforcement, the coefficient of LR_stock is significant at the 1% level and is 0.1929, that is to say, for every 1% increase in the legislative regulation, farmers' animal epidemic prevention and control input increases by 0.1929%. Meanwhile, the coefficient of LR_stock is not significant in regions with low law enforcement. It suggests that the input-enhancing effect of legislative regulation is greater in regions with high law enforcement than in regions with low law enforcement. According to the *p*-value of the coefficient difference, the positive effect of legislative regulation varies significantly in regions with different law enforcement practices. This means that local law enforcement does play a key role in contributing to the effect of legislative regulation.

**Table 7:** Heterogeneity analysis.

	**(1)**	**(2)**
	**High law enforcement**	**Low law enforcement**
**Variables**	**Input**	**Input**
LR_stock	0.1929^***^	0.0894
	(0.0320)	(0.0563)
Constant	2.3186	9.0742^***^
	(3.0762)	(2.8575)
Province FE	Yes	Yes
Year FE	Yes	Yes
CV	Yes	Yes
*p*-value	0.0000	
*N*	119	102

The standard errors are reported in the brackets. ^***^, ^**^, and ^*^ represent significance at the 1%, 5%, and 10% levels. CV represents the control variables. Yes represents all the control variables are added to the model. P-value of coefficient difference is obtained by the Chow test of interaction term model. The regression coefficients presented are significant at the 1% level, while the regression coefficients not presented are significant at either the 1%, 5%, or 10% level.

In addition, an intriguing finding is uncovered that provinces with a greater number of neighboring regions exhibit a stronger synergistic effect between legislative regulation and law enforcement in boosting farmers' animal epidemic prevention input.^23^ Areas with numerous neighbors face higher cross-area epidemic risks (64), which may amplify the marginal effect of law enforcement due to inter-jurisdictional externality internalization. Stricter local law enforcement not only reduces local epidemic risks but also mitigates spillover impacts on neighboring regions, thus amplifying the overall benefits of law enforcement.

The basic guideline for constructing the socialist legal system with Chinese characteristics is to have laws to follow and to enforce them strictly. Our study indicates that local law enforcement on animal epidemic prevention and control does play an important role in the effect of textual legislation. The integration of textual legislation and enforcement practice on pig epidemic prevention and control further enhances the promotion effect of legislative regulation. Having laws to follow and enforce them strictly complements each other. Laws and regulations enacted through legislative activities are the basis and prerequisite for ensuring strict law enforcement in the process of the rule of law; strict law enforcement is the focus of comprehensively promoting the rule of law and is the key to maintaining the authority and dignity of the law (65, 66). Since the promulgation and implementation of the Animal Husbandry Law in 2005, China has used the rule of law to promote the transformation and upgrading of the husbandry industry. The National People's Congress Standing Committee has twice carried out law enforcement inspections on this law. According to law enforcement reports, the animal epidemic prevention system is understaffed. After a new round of institutional reform, some county-level animal husbandry and veterinary departments have reduced their on-the-job personnel by more than 20%, and 65% of them have part-time jobs, even with only one animal husbandry and veterinary management personnel in some provinces.^24^ It fully illustrates the imbalance and importance of the actual enforcement intensity of animal epidemic prevention and control.

## 5 Conclusion

This study examines the impact of legislative regulation on farmers' animal epidemic prevention and control input. The main findings are as follows. First, legislative regulation has significantly increased farmers' animal epidemic prevention and control input. Farmers of different farm scales respond differently to the legislative regulation, with medium-scale farmers inputting the most in epidemic prevention and control, followed by small-scale farmers, free-range farmers, and no significant response from large-scale farmers. Second, the effect of legislative regulation on animal epidemic prevention and control input varies noticeably due to different legal hierarchies: local administrative rules > central-level administrative regulations and divisional regulations > local regulations. Third, heterogeneity analysis reveals that the input-enhancing effect of legislative regulation has been further strengthened by the integration of textual legislation and enforcement practice. Specifically, the positive effect of legislative regulation is only significant in regions with high law enforcement.

Some policy implications are obtained. First, the government should increase the law popularization and enhance farmers' awareness of legislative regulation. The government could fully understand the difficulties and realistic needs of farmers in receiving and understanding the laws and regulations on pig epidemic prevention and control through the visits and research activities of local animal epidemic prevention supervision functionaries and accordingly explore more feasible and diversified epidemic prevention and control mechanisms. In addition, it is necessary to adjust the intensity of legislative regulation based on farm scale. Second, the government should attach importance to the differentiated application of laws and regulations, assess pig development situation in various regions, and scientifically and accurately set up appropriate pig epidemic prevention and control laws and regulations. They should flexibly apply local regulations and formulate characteristic and differentiated animal epidemic prevention and control measures that are compatible with local epidemic prevention and control conditions, rather than a “one-size-fits-all” approach. Third, it is necessary to strengthen the implementation of animal epidemic prevention laws and regulations, improve the administrative capacity of supervising agencies for animal epidemic prevention and control, and standardize animal epidemic prevention law enforcement procedures, to achieve a two-pronged situation of “having laws to abide by” and “strict law enforcement” in animal epidemic prevention and control. To implement, joint regional animal prevention and control for neighbor-dense areas is also recommended.

There may be some limitations in this study. First of all, legislative regulation is a comprehensive concept covering legislation, law enforcement, and judiciary (67). Measuring it by textual legislation alone may underestimate its effect. Further research on building a complete legislative regulation index system for animal epidemic prevention and control might be more persuasive. Second, since the public data on medical and animal epidemic prevention expenditure are only available at the provincial level, there is a lack of micro-level farmers' motivation-decision response process. In the subsequent study, the latest literature and data will be tracked to supplement and improve accordingly.

## Data Availability

The original contributions presented in the study are included in the article/supplementary material, further inquiries can be directed to the corresponding author.

## Footnotes

^1^Measuring animal epidemic prevention and control behavior by a specific epidemic prevention and control measure may lead to two problems. First, in the process of raising pigs, farmers may have different priorities for animal epidemic prevention and control. For example, some regions pay attention to pre-prevention, while others focus on post-control. If a specific epidemic prevention and control measure is used to measure the behavior, it is likely to induce measurement errors. Second, when using whether to implement animal epidemic prevention and control measures to measure animal epidemic prevention and control behavior, it is easy to neglect the difference in the intensity of animal epidemic prevention and control.

^2^ https://www.stats.gov.cn/

^3^Animal Epidemic Prevention Law of the People's Republic of China, http://society.people.com.cn/n1/2021/0219/c1008-32031242.html.

^4^ https://www.agdata.cn/

^5^ https://www.pkulaw.com/

^6^ https://navi.cnki.net/knavi/yearbooks/YZGRL/detail?uniplatform=NZKPT&language=chs

^7^ https://navi.cnki.net/knavi/yearbooks/YRFTU/detail?uniplatform=NZKPT&language=chs

^8^ https://navi.cnki.net/knavi/yearbooks/YZZZD/detail?uniplatform=NZKPT&language=chs

^9^ https://navi.cnki.net/knavi/yearbooks/YZGXM/detail?uniplatform=NZKPT&language=chs

^10^ https://caaa.cn/

^11^ https://www.stats.gov.cn/sj/ndsj/

^12^ https://www.moa.gov.cn/gk/sygb/

^13^First, we do not only count the legislation for preventing and controlling specific pig epidemics, but we also keep laws and regulations that regulate the prevention and control of animal epidemics in general, as well as general laws and regulations referring to farm construction, implementation measure, and technical standard. The reason for this is that pathogens are transmitted by only a few routes, and it is possible to take effective action even if there is a gap in our knowledge of a certain epidemic. The animal epidemic prevention regulation can provide a reference for effective epidemic prevention. Second, according to the Legislation Law of the People's Republic of China, the animal epidemic prevention legislation contains seven types of laws and regulations, including law, administrative regulation, divisional regulation, local regulation, local government rule, local normative document, and local working document. Finally, the main pig epidemics comprise foot-and-mouth disease, swine vesicular disease, swine fever, porcine reproductive and respiratory syndrome, porcine cysticercosis, anthrax, swine erysipelas, swine plague, and African swine fever.

^14^Following the research logic of “having laws to abide by—strict law enforcement”, this study focuses on the effect of the text legislation of “having laws to abide by” on animal epidemic prevention and control input. The subsequent heterogeneity analysis emphasizes the further promotion effect of the integration of “having laws to abide by” and “strict law enforcement” on animal epidemic prevention and control input. So, only the cumulative number of laws and regulations is used as a proxy variable for legislative regulation.

^15^The selection of the one-period lagged education level of rural households is grounded in three key considerations. Theoretically, education's impact on agricultural behavior exhibits a temporal lag as farmers require time to internalize knowledge and apply it to production cycles. Education enhances cognitive skills, technology adoption, and risk management incrementally, aligning with the inertial nature of agricultural decision-making that relies on accumulated knowledge reserves. Empirically, data characteristics confirm sufficient temporal variation in education levels (SD = 0.3930, CV = 5.08%, Within SD = 0.2812), while correlation analysis reveals the strongest association between epidemic prevention inputs and one-period lagged education (*r* = 0.4449 vs. 0.4422 current-period and 0.3853 two-period lag). Methodologically, the xtgls model comparison demonstrates superior performance of the one-period lag specification through minimized pseudo-information criteria (Pseudo-AIC=-855.30, Pseudo-BIC = −780.54), consistent with established practices in agricultural productivity studies. This approach simultaneously addresses endogeneity concerns while capturing education's delayed yet cumulative effects on behavioral change.

^16^The “+1” transformation is a common practice in econometrics and statistics when dealing with non-negative data that includes zeros. It allows us to work with the logarithm of the variable while preserving the integrity of the data.

^17^The modified Wald test value is 80.19, the Woodridge test value is 10.817, and the Pesaran test value is 3.631 with a *p*-value of 0.0003.

^18^Source: https://www.chinanews.com.cn/sh/2019/08-20/8931863.shtml.

^19^“Neighboring provinces” are defined as provinces bordering each other.

^20^ https://lawnew.cnki.net/kns/brief/result.aspx?dbPrefix=CLKD

^21^Policy implementation and production adjustments inherently involve time lags due to information transmission delays and adaptive decision-making processes. As policymakers signal regulatory changes (e.g., enhanced animal epidemic controls), farmers require time to interpret policy credibility, modify production techniques, and overcome the “wait-and-see” inertia characteristic of agricultural cycles. These real-world response delays align with the biological constraints of livestock production cycles and institutional friction in policy enforcement. In addition, this approach follows established econometric practice in panel data analysis (68, 71), where lagged variables help address potential reverse causality and temporal mismatch between policy signals and observable outcomes. Therefore, we conduct a robustness check by using the one-period lagged term of the core independent variable as a proxy for the core explanatory variable.

^22^Local administrative rules here include local government rules, local normative documents, and local working documents.

^23^See Appendix for details.

^24^Source: https://www.sohu.com/a/484491008_29936.

## Appendix

**Table A1:** Impact of the number of neighbors on the moderating effect of law enforcement.

**Variables**	**Input**
LR_stock	0.3135^***^
	(0.0366)
Enforce	1.2828^***^
	(0.1909)
Neighbor	1.1988^***^
	(0.1249)
LR_stock × Enforce × Neighbor	0.1348^***^
	(0.0271)
Constant	0.5905
	(1.4991)
Province FE	Yes
Year FE	Yes
N	221

Enforce = 1 for provinces with high law enforcement intensity and Enforce = 0 for low-enforcement regions. Neighbor = 1 for provinces with a neighboring region count above the median and Neighbor = 0 otherwise. The standard errors are reported in the brackets. ^***^, ^**^, and ^*^ represent significance at the 1%, 5%, and 10% levels. CV represents the control variables. Yes represents all the control variables are added to the model. The regression coefficients presented are significant at the 1% level, while the regression coefficients not presented are significant at either the 1%, 5%, or 10% level.
